# The ward atmosphere important for the psychosocial work environment of nursing staff in psychiatric in-patient care

**DOI:** 10.1186/1472-6955-10-12

**Published:** 2011-06-16

**Authors:** Hanna Tuvesson, Christine Wann-Hansson, Mona Eklund

**Affiliations:** 1 Faculty of Health and Society, Malmö University, SE-205 06 Malmö, Sweden; 2 Department of Health Sciences, Lund University, PO Box 157, SE-221 00 Lund, Sweden

## Abstract

**Background:**

The nursing staff working in psychiatric care have a demanding work situation, which may be reflected in how they view their psychosocial work environment and the ward atmosphere. The aims of the present study were to investigate in what way different aspects of the ward atmosphere were related to the psychosocial work environment, as perceived by nursing staff working in psychiatric in-patient care, and possible differences between nurses and nurse assistants.

**Methods:**

93 nursing staff working at 12 general psychiatric in-patient wards in Sweden completed two questionnaires, the Ward Atmosphere Scale and the QPSNordic 34+. Data analyses included descriptive statistics, the Mann-Whitney *U*-test, Spearman rank correlations and forward stepwise conditional logistic regression analyses.

**Results:**

The data revealed that there were no differences between nurses and nurse assistants concerning perceptions of the psychosocial work environment and the ward atmosphere. The ward atmosphere subscales Personal Problem Orientation and Program Clarity were associated with a psychosocial work environment characterized by Empowering Leadership. Program Clarity was related to the staff's perceived Role Clarity, and Practical Orientation and Order and Organization were positively related to staff perceptions of the Organizational Climate.

**Conclusions:**

The results from the present study indicate that several ward atmosphere subscales were related to the nursing staff's perceptions of the psychosocial work environment in terms of Empowering Leadership, Role Clarity and Organizational Climate. Improvements in the ward atmosphere could be another way to accomplish improvements in the working conditions of the staff, and such improvements would affect nurses and nurse assistants in similar ways.

## Background

The nursing staff working in psychiatric care have a demanding work situation, which may be reflected in how they view their psychosocial work environment and the ward atmosphere. Despite an extensive body of research in the field of psychosocial work environment [1-3] and in that of the ward atmosphere [4-6], there is little research that investigates the relationship between these two phenomena in psychiatric care. The psychosocial work environment has to do with the staff's working conditions, including organizational and work characteristics [7], while the ward atmosphere reflects the milieu in which the care takes place and patient - staff relationships are developed [5]. An understanding of how the nursing staff's perceptions of the ward atmosphere are related to the psychosocial work environment may contribute to new ways of improving their work conditions. Should a relationship be found, changing the ward atmosphere might be an alternative pathway for affecting the staff's psychosocial work environment.

Health care systems in many industrialized countries, such as Sweden, have undergone major changes in order to improve efficiency and to reduce the number of hospital beds [8]. Still, in-patient care plays a major role in the care and treatment of patients with psychiatric problems [9]. The major changes in psychiatric care have affected the nursing staff's personal situation at work, creating work in complex environments and putting organizational pressure and high demands on the staff [10].

The psychosocial work environment can be described as a multifaceted and complex phenomenon and may be framed according to three levels; the Task level involving aspects of control, role expectations, and job demands, the Social and Organizational level involving aspects such as social interaction, communication, leadership and organizational culture, and finally the Individual level comprising aspects of, for example, commitment and work motives [7]. The psychosocial work environment is crucial for the well-being and health of nursing staff working in psychiatric care and has been linked to the staff's perceived burnout, psychological distress and stress [11-14]. Moreover a number of work environmental factors have been linked to the staff's absence due to illness [12] and to perceived job satisfaction [15-17]. Not only is the psychosocial work environment in psychiatric care important for the staff, it may also be reflected in the delivery of care and the relationship between the staff and the patients. Studies have, for example, found that the working conditions of psychiatric nursing staff are closely related to the quality of care, as perceived by patients [2] and that in order to develop an effective treatment setting, the work environment should satisfy the staff [5]. The staff's working conditions may thus also be an important factor that influences the patients and the care. Investigating factors that can influence how nursing staff perceive their psychosocial work environmental conditions may be of importance for improving their working conditions, and the care they deliver. As indicated above, issues related to the care of and relationships to patients, here framed as the psychosocial ward atmosphere, may be one such influential factor.

The ward atmosphere has been described by Eklund and Hansson [18] as a phenomenon shaped by the social structures and social interaction in the caring environment. Moos [5] expressed early on the necessity of putting an emphasis on the ward atmosphere and its influence on patients and staff in psychiatric and substance abuse programs. He described the ward atmosphere by means of three dimensions; Relationships dimension, Personal growth dimension, and System maintenance dimension. The Relationship dimension involves aspects of the quality of personal relationships, involvement and support in the ward. The Personal growth dimension captures the level of encouragement for personal change and development among patients. Finally, the System maintenance dimension emphasizes how well ordered and organized a ward is [5].There is a large body of research about the ward atmosphere in psychiatric care and many studies have examined patients' perceptions. Results have shown that the ward atmosphere is important for treatment outcome [6,18] and patient satisfaction [19]. Studies that have included the staff found that these tended to view the ward atmosphere more favorably than the patients did [20-22]. Moreover, the ward atmosphere factor Order and Organization has been found to support high morale among nursing staff in psychiatric in-patient care [23]. Thus, there are some indications that certain aspects of the ward atmosphere may be related to the psychosocial work environment of the staff, but the relationship is far from clarified.

Different categories of staff may perceive their psychosocial work environment and the ward atmosphere differently, possibly due to them not having the same types of duties and responsibilities. Although nurses and nurse assistants work side by side, it is important to consider their different roles and thus possible differences in perceptions regarding the psychosocial work environment and the ward atmosphere. A few previous comparative studies have been made. Dallender and associates [24], for example, compared psychiatric nurses and psychiatrists regarding their perceptions of the physical work environment. They found that the nurses reported more heavy lifts and a noisier environment, while no significant differences were found regarding their perception of the social work climate and mental well-being. Comparative studies of nurses and nurse assistants in psychiatric care focusing on the ward atmosphere and perceived psychosocial work environment are rare. Those studies that have compared these staff groups have focused on stress and burnout and found no differences in those respects between nurses and nurse assistants [25,26]. Small differences could, however, be seen between stressors: the main stressors for nurses was lack of resources [25] and high work demands [26], while for the nurse assistants it was client-related difficulties [26] and social relations [27]. No studies that specifically investigate differences in perceptions of the ward atmosphere among nurses and nurse assistants in psychiatric in-patient care seem to have been performed. It is important to build an understanding of whether these two occupational groups' perceptions of the ward atmosphere and the psychosocial work environment differ, in order to establish what should be the focus of workplace improvements and whether these two groups would benefit from the same improvements.

As mentioned above, the ward atmosphere may be an important influential factor for the psychosocial work environment of the nursing staff, and it could also be used as a platform to indentify and find solutions for psychosocial work environmental problems. No studies appear to have investigated whether there is a relationship between the ward atmosphere and the psychosocial work environment, as perceived by the nursing staff in psychiatric in-patient care. The aims of the present study were thus to investigate in what way aspects of the ward atmosphere were related to the psychosocial work environment as perceived by nursing staff working in psychiatric in-patient care, while also paying attention to individual characteristics such as age and length of employment. A further aim was to describe if there were any differences between how nurses and nurse assistants perceived the psychosocial work environment and the ward atmosphere.

## Methods

The study was a descriptive cross-sectional survey of nursing staff working in general in-patient psychiatric care in the south of Sweden.

### Selection procedure and subjects

This study complied with stipulations in the Swedish act regulating research ethics and the principle of informed consent was applied (approval was obtained from the Regional Ethical Review Borad in Lund, dnr 380/2008). In Sweden, psychiatric care is organized in geographical areas, and the units in the present study were each responsible for the in-patient care of a specific area. The wards selected for the present study were psychiatric in-patient wards in the south of Sweden, and all of the 12 wards that had been approached agreed to participate. All nurses and nurse assistants who worked at one of those wards for a minimum of two months and worked daytime were invited to take part in the study. All participants received written information about the aim of the study and about the principles of confidentiality and voluntary participation. An information meeting was then held at all 12 wards and the questionnaires were administered immediately after. Those staff members who were not present at the information meeting received the questionnaire together with the written information from the ward manager. The questionnaires were then put in sealed envelopes to guaranty anonymity, and subsequently posted back to the first author.

No previous research existed that could provide a correct basis for a power calculation. An estimation based on reasoning around effect sizes was, however, made. The researchers aimed for a medium effect size of 0.5 in accordance with Cohen [28]. A sample of 100 participants was thus needed for an effect size with 80% power at p < 0.05 [29]. The questionnaires were distributed, to 179 nursing staff (70 nurses & 109 nurse assistants). Completed questionnaires were received from 93 participants (response rate = 52.3%), 38 nurses (response rate = 54.3%) and 55 nurse assistants (response rate = 50.5%), thus, the response rates were similar in both occupational groups. The proportion of nurses was 39% among the total staff to which the questionnaires were distributed; it was similar, 41%, in the sample of respondents. Similarly, the mean age was similar in the total staff group (48.9 years) and the study sample (48 years). By the 93 participants the study did not quite reach the desired sample size. Characteristics of the participants are presented in Table 1. The majority were females and had permanent employment on the ward. The participants were between 21 and 65 years old and were both nurses (*n = *38) and nurse assistants (*n *= 55). Their average length of experience in psychiatric care was 18 years and the average length of employment at the actual ward was 9 years

**Table 1:** Characteristics of the sample (*n *= 93).

Characteristics (*n*: 93)	
Sex (female/male) ^1)^	72/20
Mean age (range, SD) ^2)^	48 (21 - 65, 11)
Profession (reg. nurse/nurse ass.)	38/55
Employment (permanent/temporary) ^3)^	80/8
Mean years on actual ward (range, SD)	9 (0.17 - 30, 8)
Mean years of experience in psychiatry (range, SD) ^1)^	18 (0.17 - 41, 13)

^1) ^One missing value

^2) ^Two missing values

^3) ^Five missing values

### Instruments

#### QPSNordic 34+

The QPSNordic 34+ consists of 37 questions and is a short version of the General Nordic Questionnaire for Psychological and Social Factors at Work (QPSNordic). The respondents rate their answers on a five-point scale, ranging from Very seldom or never (1) to Very often or always (5) [7,30,31]. The QPSNordic has shown to be reliable, with Cronbach's alpha values ranging from 0.62 to 0.86 [30]. However, to the best of our knowledge, the psychometric properties of the QPSNordic 34+ do not seem to have been previously tested, which led us to investigate possible subscales in the short version. The criterion for an acceptable Cronbach's alpha value of > 0.70 was set. Subscales corresponding to the aspects and factors of the full QPSNordic were tested according to this criterion and five acceptable subscales were identified. These were: Empowering Leadership (2 items; Cronbach's alpha = 0.85), Role Clarity (2 items; Cronbach's alpha = 0.79), Control at Work (4 items; Cronbach's alpha = 0.72), Support from Superiors (2 items; Cronbach's alpha = 0.8), and Organizational Climate (6 items; Cronbach's alpha = 0.77). No sum score for the QPSNordic 34+ was used, since it was devised to reflect different facets of the psychosocial work environment.

#### Ward Atmosphere Scale

A revised version of the Ward Atmosphere Scale (WAS), originally developed by Moos [5], was used to characterize the ward atmosphere at the wards. This revised and updated version of the WAS comprises 83 items and is a self-rating scale where the participants give their responses on a four-point scale ranging from Totally disagree (0) to Totally agree (3). This revised WAS consists of eleven subscales, which are to be analyzed separately, and has previously been found to have acceptable internal consistency for all subscales except Autonomy [32-34]. The present study, however, only includes six subscales due to low alpha values for several of the subscales when the internal consistency was investigated. The alpha values of the included subscales: Involvement, Practical Orientation, Personal Problem Orientation, Angry and Aggressive Behavior, Order and Organization, and Program Clarity, ranged between 0.53 and 0.69.

### Statistical analysis

Descriptive statistics for characteristics of the participants and response distributions on the scales were calculated. The nonparametric Mann-Whitney *U*-test was used to test differences between groups and Spearman rank correlations were used to analyze associations between variables. The Mann-Whitney *U*-test and the Spearman rank correlations served to identify factors that could be used as independent variables in logistic regression analyses. The latter were performed in order to calculate multivariate relations and assess which staff characteristics and ward atmosphere variables contributed to explaining variation in the psychosocial work environment. Each of the five work environmental aspects was set to be the dependent variable in relation to ward atmosphere and individual characteristics. All continuous variables were transformed into categorical ones by dichotomizing the total group according to a median cut on each variable. A high and a low group were thus created for all variables. Forward stepwise conditional logistic regression analyses were carried out. The p-level for entering an independent variable in those analyses was set at *p *= < 0.1. For all other analyses a significance level of 0.05 was applied. The analyses were performed with the statistical software package SPSS (version 17.0).

## Results

Table 2 shows the mean scores for the ward atmosphere and psychosocial work environment subscales for nurses and nurse assistants separately. The result of the Mann-Whitney *U*-test showed no significant differences between the staff groups concerning any of the psychosocial work environment and ward atmosphere aspects.

**Table 2:** Mean scores for the ward atmosphere and psychosocial work environment

	Nurses (*n*: 38)	Nurse assistants (*n: *55)	**P-value**^**1**^
	(SD; Min-Max)	(SD; Min-Max)	
**Ward atmosphere subscales**			
Involvement	1.53 (0.27; 1-2.1)	1.44 (0.33; 0.6-2)	0.224
Practical Orientation	1.77 (0.34; 1.14-2.57)	1.80 (0.41; 0.86-3)	0.768
Personal Problem	2.19 (0.32; 1.5-2.67)	2.05 (0.36; 1.33-2.67)	0.070
Orientation			
Angry and Aggressive Behavior	1.08 (0.41; 0.25-2)	1.07 (0.41; 0.33-2.44)	0.686
Order and Organization	1.58 (0.4; 0.8-2.3)	1.67 (0.33; 0.6-2.3)	0.191
Program Clarity	1.84 (0.36; 0.89-2.56)	1.85 (0.34; 1.06-2.67)	0.944
			
**Psychosocial work environmental subscales**			
Empowering Leadership	3.30 (0.93; 1-5)	3.29 (1.04; 1-5)	0.911
Role Clarity	3.61 (0.9; 1-5)	3.87 (0.78; 2-5)	0.145
Control at Work	3.09 (0.63; 1.75-4.75)	2.89 (0.8; 1-4.25)	0.307
Support from Superiors	1.73 (0.39; 1-2.5)	1.68 (0.5; 1-3)	0.470
Organizational Climate	3.08 (0.55; 1.67-4.17)	3.07 (0.8; 1-4.83)	0.934

^1)^Mann-Whitney *U*-test.

The bivariate analyses are presented in Table 3. Empowering Leadership had statistically significant correlations with all ward atmosphere subscales except Angry and Aggressive Behavior, and Role Clarity was associated with Practical Orientation, Order and Organization and Program Clarity. The subscale Support from Superiors showed statistically significant correlations with two of the ward atmosphere scales, Involvement and Practical Orientation. Furthermore, there were significant correlations between Organizational Climate and all ward atmosphere subscales except Personal Problem Orientation. There were no statistically significant correlations between the psychosocial work environmental factor of Control at Work and any of the ward atmosphere subscales. There were also no statistically significant association between any of the individual characteristics and the psychosocial work environment subscales.

**Table 3:** Correlations between individual characteristics, ward atmosphere factors and psychosocial work environment subscales.

Individual characteristics, and ward atmosphere subscales	Empowering Leadership	Role Clarity	Control at Work	Support from Superior	Organizational Climate
**Age**	-.158	.151	.009	.113	.067
**Experience on actual ward**	.047	.190	.062	.085	.148
**Experience in psychiatry**	-.139	.191	-.017	.132	.063
**Involvement**	.338**	.198	.131	.256*	.391**
**Practical Orientation**	.314**	.234*	-.065	.241*	.376**
**Personal Problem Orientation**	.216*	-.036	-.113	.046	.117
**Angry and Aggressive Behavior**	-.179	-.087	-.127	-.066	-.250*
**Order and Organization**	.254*	.374**	.159	.024	.414**
**Program Clarity**	.293**	.271**	.041	.186	.457**

**p <.01, *p <.05

Variables that had shown a relationship of p < 0.1 with the respective psychosocial work environment subscales (cf. Table 3) were entered in the logistical regression models. Table 4 presents the findings. The analysis concerning Empowering Leadership revealed two ward atmosphere subscales of significance: Program Clarity and Personal Problem Orientation. Belonging to the high group on those two ward atmosphere subscales more than doubled the likelihood of perceiving a high level of Empowering Leadership. Role Clarity was associated with one significant factor, and the analysis indicated that those who belonged to the high group on Program Clarity had a more than threefold chance of being in the high group of perceived Role Clarity. Finally, the analysis regarding Organizational Climate resulted in two significant factors. Being in the high group regarding Order and Organization increased the likelihood of belonging to the high group of Organizational Climate by more than four times, and perceiving a high level of Practical Orientation increased that likelihood further, as indicated by the odds ratio close to three.

**Table 4:** Ward atmosphere subscales of importance for aspects of the work environment.

Dependent variable	Independent variable	p	OR	95% CI for OR
Empowering Leadership	Program Clarity Personal	0.025	2.695	1.132 - 6.419
	Personal Problem Orientation	0.040	2.479	1.041 - 5.901
Role Clarity	Program Clarity	0.007	3.248	1.371 - 7.693
Organizational Climate	Order and Organization	0.004	4.288	1.608 - 11.430
	Practical Orientation	0.025	2.982	1.150 - 7.734

Note: analyses based on a forward stepwise conditional logistic regression (*p *= < 0.05)

The significant ward atmosphere variables accounted for 14.7% of the variance (Nagelkerke *R*^2^) in Empowering Leadership, 10.4% in Role Clarity, and 23.3% in Organizational Climate. All three models exhibited acceptable goodness-of-fit (Hosmer-Lemeshow test, *p *> 0.05).

## Discussion

The findings clearly indicated an association between how the nursing staff rated the ward atmosphere and how they perceived their psychosocial work environment, although the correlations were small to moderate in size [28]. This indicates that the ward atmosphere and the psychosocial work environment were related but separate phenomena. The data also revealed that there were no differences between nurses and nurse assistants concerning perceptions of the psychosocial work environment and the ward atmosphere.

Two of the ward atmosphere subscales, Personal Problem Orientation and Program Clarity, were important for the participants' perception of Empowering Leadership. High ratings on those two aspects of ward atmosphere were associated with perceiving a high level of Empowering Leadership. The latter assesses the degree to which the leadership stimulates encouragement and empowerment to take part in decision-making and developments. The importance of leadership for the staff's working conditions is well recognized. Empowering leadership may increase an individual's feelings of organizational justice, respect, and trust in management [35], and leadership empowerment has also been related to job satisfaction [35]. Furthermore, Kanter [36] suggests that employees' attitudes would improve along with an increase in organizational effectiveness if structured empowering is used. This indicates that it is important to increase leadership empowerment and a possible way to facilitate this could be to improve the ward atmosphere factors of Personal Problem Orientation and Program Clarity. Personal Problem Orientation assesses the degree of encouragement for the patients to freely and openly express and talk about feelings and personal problems. Program Clarity involves how stabile, evident and clear the treatment structure, rules and expectations are at the ward [5]. It might be that the encouraging nature of Personal Problem Orientation is also important to the nursing staff, in terms of increasing the leadership empowerment. However, no previous research seems to exist concerning the relationship of these aspects, and there are thus no studies that can confirm that assumption. Furthermore, the clarity aspect regarding treatment structure, rules and expectations embedded in Program Clarity may stimulate an encouraging and empowering leadership style by enabling managers to involve the staff in decisions and developments concerning ward and treatment issues. The relationship may, however, be circular. The ward atmosphere may be important for empowering leadership, but improvements in the ward atmosphere may also, through for example staff training [37], be initiated by an empowering leadership.

The findings of the present study also suggest that Program Clarity was related to the nursing staff's perceived Role Clarity. It seems reasonable to assume that a high level of Program Clarity, with a clear treatment structure and clarity of expectations, would increase the Role Clarity as appraised by the nursing staff. Role clarity has been found to be an important aspect in relation to perceived stress among psychiatric staff [13,14,38]. To find ways to increase the Role Clarity, as perceived by the nursing staff, thus seems to be important. A possible way to accomplish this would be to concentrate on both the psychosocial work environmental aspects and the ward atmosphere and thus work on two fronts in order to make improvements.

Practical Orientation and Order and Organization explained almost one fourth of the variance in Organizational Climate. The Organizational Climate, as measured in the present study, taps aspects such as perceived levels of encouragement, support and comfort in the organization. It also involves levels of appraised communication within teams and between group members. Social support from colleagues is an important factor of the psychosocial work environment [39]. In the present study, ratings of the ward atmosphere subscale of Practical Orientation and Order and Organization were positively related to nursing staff perceptions of the Organizational Climate. Practical Orientation denotes the degree to which the staff and the ward activities guide the patients towards problem solving, both during the latter's admission to the ward and outside of it. Order and Organization concerns the staff's ability to keep scheduled appointments with patients and the patient's ability to follow daily schedules and treatment plans [5]. No previous studies seem to have focused on the relationship between these aspects, but one could speculate that the identified association between ward atmosphere factors and the nursing staff's perception of the psychosocial work environment might indicate that high levels of Practical Orientation and Order and Organization could stimulate staff communication and increase support between co-workers. Furthermore, with high levels of Order and Organization the staff might be more likely to keep arrangements and appointments with the patients, and do this on time. It is possible that this may lead to a reduction in tension between colleagues, as well as to improved communication and support. It may also be the other way around, i.e. that a beneficial Organizational Climate promotes Practical Orientation and Order and Organization. Either way, the relationships between these phenomena indicate that also involving ward atmosphere issues could be a more effective approach, when addressing a psychosocial work environment problem in this area.

The study did not identify any differences between nurses and nurse assistants in terms of perceived psychosocial work environment or ward atmosphere. No previous studies seem to have addressed those phenomena, however, studies have found similar results concerning perceived stress and burnout among nurses and nurse assistants [25,26]. Although these staff groups tend to have somewhat different work responsibilities, they seem to perceive the psychosocial work environment and ward atmosphere in similar ways. It can thus also be argued that any improvements in the psychosocial work environment and the ward atmosphere would be beneficial to both staff groups.

The descriptive results from the present study indicate that the participants appeared to rate the psychosocial work environment and ward atmosphere as moderately high for most of the subscales. However, the nursing staff perceived the Support from Superiors as rather low. In addition, no ward atmosphere variables were found to be related to this psychosocial work environment aspect according to the logistic regression. The significant bivariate association found in the correlational analysis disappeared after dichotomizing for the logistic regression, in which some of the variation in the variables was lost. It is possible that Support from Superiors is mainly contingent on other factors, not addressed in this study. It is also possible, that the leadership style on the wards in the present study emphasized empowering aspects and role clarity, and that leadership support was less emphasized.

There are some reservations about these findings. The response rate was lower than desired (52.3%), despite several reminders, and it is possible that there was a selection bias, in turn possibly influencing the results. Still, the present sample seemed to be representative of the staff working at the 12 units in terms of mean age and the distribution of nurses/nurse assistants. The reliability of the instruments used for the data collection was another problem of this study, where five of the WAS subscales had to be excluded from the analysis due to low Cronbach's alpha values (<0.5). Similar problems have been reported previously, especially concerning the factor structure of WAS [40,41]. These methodological issues have also been reported for the revised version [32,33]. According to Røssberg and Friis [33], a Cronbach's alpha ≥0.5 is acceptable for comparing ward means [33], and since the present study included only aggregated data, the WAS subscales were kept for analysis if they reached that value. The instrument used to assess psychological and social factors at work, QPSNordic 34+, has to our knowledge not been formally tested. In the present study variables were formed on the basis of areas and scales identified in the full version of QPSNordic. With this in mind, further studies are warranted in order to establish subscales of the QPSNordic 34+. Although four of the ward atmosphere variables contributed significantly to the psychosocial work environment variables of Empowering Leadership, Role Clarity, and Organizational Climate, the amount of explained variance was limited. Especially concerning Empowering Leadership (10.4%) and Role Clarity (14.7%), the findings indicate that contextual variables not included in this study, such as the work load and the physical milieu, may be of importance for the psychosocial work environment. Besides, personal characteristics, such as self-efficacy and self-mastery, might mediate the relationship between the ward atmosphere and the way the psychosocial work environment is perceived, but this remains to be studied. This study fulfilled the purpose of specifically focusing on how ward atmosphere factors and the psychosocial work environment were related, which opens up for studies of more complex relationships in future research.

## Conclusions

In conclusion, the results from the present study indicate that several ward atmosphere subscales were related to aspects of the psychosocial work environment in terms of Empowering leadership, Role clarity and Organizational climate. The clarity of the treatment programs, the encouragement of patients, and the staff's focus on feelings and personal problems among the patients were found to be important ingredients embedded in the ward atmosphere. These were factors that were shown to influence the nursing staff's psychosocial work environment. Improvements in the ward atmosphere could be another way to accomplish improvements in the working conditions for the staff, in terms of psychosocial work environment, and the ward manager may have an important role in this respect. Improvement and changes in the ward atmosphere, and also in the psychosocial work environment, would seemingly affect nurses and nurse assistants in similar ways. Specifically, the findings indicate that developing and improving the ward atmosphere by clarifying the ward and treatment/caring structure and regimes, and enhancing activities that guide the patients in personal and practical problem solving, could pave the way for a better psychosocial work environment. This study was limited to the relationships between the ward atmosphere and the psychosocial work environment. The results may serve as a basis for generating hypotheses regarding more complex relationships between potentially influencing factors and aspects of the psychosocial environment. These can, for example, include buffering factors like peer support and personal characteristics such as feeling that one is in control of one's life situation.

## Competing interests

The authors declare that they have no competing interests.

## Authors' contributions

Each author has contributed to the authorship of the present study. HT and ME have contributed to the initial design of the study and together with CWH created the final design. HT has conducted the data collection and has been the primary author of the study. The data has been analyzed by all three authors and ME and CWH have actively contributed to the drafting and revision of the article and approved the final version of this article. All authors have read and approved the final manuscript.

## Pre-publication history

The pre-publication history for this paper can be accessed here:

http://www.biomedcentral.com/1472-6955/10/12/prepub
